# Risk‐dependent curability of radiotherapy for elderly patients with early‐stage extranodal nasal‐type NK/T‐cell lymphoma: A multicenter study from the China Lymphoma Collaborative Group (CLCG)

**DOI:** 10.1002/cam4.1849

**Published:** 2018-10-24

**Authors:** Bo Chen, Su‐Yu Zhu, Mei Shi, Hang Su, Ying Wang, Xia He, Li‐Ming Xu, Zhi‐Yong Yuan, Li‐Ling Zhang, Gang Wu, Bao‐Lin Qu, Li‐Ting Qian, Xiao‐Rong Hou, Fu‐Quan Zhang, Yu‐Jing Zhang, Yuan Zhu, Jian‐Zhong Cao, Sheng‐Min Lan, Jun‐Xin Wu, Tao Wu, Shu‐Nan Qi, Yong Yang, Xin Liu, Ye‐Xiong Li

**Affiliations:** ^1^ State Key Laboratory of Molecular Oncology and Department of Radiation Oncology National Cancer Center/National Clinical Research Center for Cancer/Cancer Hospital, Chinese Academy of Medical Sciences (CAMS) and Peking Union Medical College (PUMC) Beijing China; ^2^ Center for Cancer Precision Medicine CAMS and PUMC Beijing China; ^3^ National Institute of Biological Sciences Collaborative Innovation Center for Cancer Medicine Beijing China; ^4^ Department of Radiation Oncology Hunan Cancer Hospital and the Affiliated Cancer Hospital of Xiangya School of Medicine Changsha China; ^5^ Department of Radiation Oncology Xijing Hospital, Fourth Military Medical University Xi'an China; ^6^ Department of Oncology 307 Hospital, Academy of Military Medical Science Beijing China; ^7^ Department of Radiation Oncology Chongqing Cancer Hospital & Cancer Institute Chongqing China; ^8^ Department of Radiation Oncology Jiangsu Cancer Hospital & Jiangsu Institute of Cancer Research Nanjing China; ^9^ Department of Radiation Oncology Tianjin Medical University Cancer Institute & Hospital, Key Laboratory of Cancer Prevention and Therapy, National Clinical Research Center for Cancer Tianjin China; ^10^ Cancer Center Union Hospital, Tongji Medical College, Huazhong University of Science and Technology Wuhan China; ^11^ Department of Radiation Oncology The General Hospital of Chinese People's Liberation Army Beijing China; ^12^ Department of Radiation Oncology The Affiliated Provincial Hospital of Anhui Medical University Hefei China; ^13^ Department of Radiation Oncology Peking Union Medical College Hospital, Chinese Academy of Medical Sciences (CAMS) and Peking Union Medical College (PUMC) Beijing China; ^14^ Department of Radiation Oncology Sun Yat‐sen University Cancer Center Guangzhou China; ^15^ State Key Laboratory of Oncology in South China Guangzhou China; ^16^ Collaborative Innovation Center for Cancer Medicine Guangzhou China; ^17^ Department of Radiation Oncology Zhejiang Cancer Hospital Hangzhou China; ^18^ Department of Radiation Oncology Shanxi Cancer Hospital and the Affiliated Cancer Hospital of Shanxi Medical University Taiyuan China; ^19^ Department of Radiation Oncology Fujian Provincial Cancer Hospital Fuzhou China; ^20^ Department of Lymphoma Affiliated Hospital of Guizhou Medical University, Guizhou Cancer Hospital Guiyang China

**Keywords:** elderly, NK/T‐cell lymphoma, prognosis, radiotherapy, risk stratification

## Abstract

**Background:**

The purpose of this study was to determine the curability of early‐stage extranodal nasal‐type NK/T‐cell lymphoma (NKTCL) in response to radiotherapy and non‐anthracycline‐based chemotherapy in elderly patients.

**Methods:**

In this multicenter study from the China Lymphoma Collaborative Group (CLCG) database, 321 elderly patients with early‐stage NKTCL were retrospectively reviewed. Patients received radiotherapy alone (n = 87), chemotherapy alone (n = 59), or combined modality therapy (CMT, n = 175). Patients were classified into low‐ or high‐risk groups using four prognostic factors. Observed survival in the study cohort vs expected survival in age‐ and sex‐matched individuals from the general Chinese population was plotted using a conditional approach and subsequently compared using a standardized mortality ratio (SMR).

**Results:**

Radiotherapy conveyed a favorable prognosis and significantly improved survival compared to chemotherapy alone. The 5‐year overall survival (OS) and progression‐free survival (PFS) were 61.2% and 56.4%, respectively, for radiotherapy compared with 44.7% and 38.3%, respectively, for chemotherapy alone (*P < *0.001). The combination of a non‐anthracycline‐based chemotherapy regimen and radiotherapy significantly improved PFS compared to combination of an anthracycline‐based chemotherapy regimen and radiotherapy (71.2% vs 44.2%, *P* = 0.017). Low‐risk patients following radiotherapy (SMR, 0.703; *P* = 0.203) and high‐risk patients who achieved PFS at 24 months (SMR, 1.490; *P = *0.111) after radiotherapy showed survival equivalent to the general Chinese population.

**Conclusions:**

Our findings indicate a favorable curability for this malignancy in response to radiotherapy and non‐anthracycline‐based chemotherapy, providing a risk‐adapted follow‐up and counsel scheme in elderly patients.

## 1 INTRODUCTION

Extranodal nasal‐type NK/T‐cell lymphoma (NKTCL) is the most common subtype of peripheral T‐cell lymphoma in China and East Asia^1^, ^2^ but is rare in Western countries.^3^ NKTCL is primarily localized in the upper aerodigestive tract (UADT), the nasal cavity and Waldeyer's ring, exhibiting an aggressive, heterogeneous clinical course.^4^, ^5^, ^6^, ^7^ Unlike diffuse large B‐cell lymphoma (DLBCL) that is primarily diagnosed at an advanced stage in older patients, NKTCL typically presents at an early stage in younger patients.^4^, ^5^, ^8^, ^9^, ^10^ Previous studies of NKTCL reported a median patient age of 40‐50 years, which is ten years younger than that of patients with DLBCL, with 14% to 43% of patients being 60 years or older.^5^, ^8^, ^9^, ^10^

Prior studies have identified patient age as an important prognostic factor in aggressive lymphoma.^11^, ^12^, ^13^, ^14^, ^15^ Age>60 years is an independent adverse factor incorporated into the international prognostic index (IPI) for DLBCL^16^, ^17^ and more recently in developed prognostic models for NKTCL.^5^, ^8^ Our previous studies using a small number of NKTCL cases at a single institution revealed favorable outcomes for children and adolescents^18^ but unfavorable prognoses for elderly patients.^19^ However, the prognostic significance and treatment outcome of elderly patients with NKTCL have not been established in large, multicenter studies.^20^, ^21^

Over the past decade, management of early‐stage NKTCL has evolved from primary chemotherapy with anthracycline‐based regimens to utilization of combined upfront radiotherapy and non‐anthracycline‐based chemotherapy.^22^, ^23^, ^24^, ^25^, ^26^ Radiotherapy has proven to be an essential component of frontline treatment strategies for early‐stage NKTCL, demonstrating a significantly higher survival rate than that of patients treated with chemotherapy alone.^10^, ^25^ Furthermore, we have previously shown that risk‐adapted therapy with radiotherapy alone for low‐risk patients and radiotherapy followed by chemotherapy for high‐risk patients resulted in favorable outcomes, with 5‐year overall survival (OS) rates between 70% and 90%.^25^ In this study, all early‐stage patients older than 60 years were classified into a high‐risk group, regardless of other prognostic factors. Nevertheless, the benefit of frontline curative‐intent radiotherapy remains unclear for elderly patients with early‐stage NKTCL^19^, ^20^ because primary radiotherapy that is translated into high cure rates could be influenced by competing risks of death related to other diseases. In addition, given the high frequency of comorbidities and diminished organ function in older patients,^12^ the safety and efficacy of an intensified, new chemotherapy regimen needs to be defined in early‐stage NKTCL.^23^, ^24^ The purpose of the present study was to examine the curability of early‐stage NKTCL in response to radiotherapy for risk‐stratified elderly patients and to compare the risk of death in such patients to that of the general Chinese population using age‐, sex‐, and calendar period‐generated mortality rates.

## 2 PATIENTS AND METHODS

### 2.1 Patient eligibility

A total of 2640 patients with newly diagnosed NKTCL treated at 16 Chinese institutions between 2000 and 2015 were analyzed from the China Lymphoma Collaborative Group (CLCG). Eligibility criteria for this study included the following: (a) patient age >60 years old; (b) stage I and II disease according to the Ann Arbor staging system; and (c) treatment with curative intent. Of the 2640 patients, 361 patients (13.7%) were >60 years old. Of the 361 elderly patients, 40 patients with advanced‐stage disease were excluded, leaving 321 patients with early‐stage disease forming the study population. This project was approved by our institutional review board and conducted in accordance with the Declaration of Helsinki.

### 2.2 Treatment

Elderly patients with early‐stage NKTCL received radiotherapy alone (n = 87), chemotherapy alone (n = 59), or combined modality therapy (CMT, n = 175), with 262 patients receiving radiotherapy in total (with or without chemotherapy). Radiotherapy included extended involved‐site field covering the primary tumor, adjacent organs, and lymph node areas.^27^, ^28^, ^29^ The median dose was 50 Gy at a dose per fraction of 1.8‐2 Gy, and 50.4% of patients received intensity‐modified radiation therapy. Of the 234 patients who received chemotherapy, 122 (52.1%) received non‐anthracycline‐based chemotherapy, including asparaginase‐, gemcitabine‐, or etoposide‐based regimens (new regimen), whereas 112 patients (47.9%) received anthracycline‐based chemotherapy, including CHOP (cyclophosphamide, doxorubicin, vincristine, prednisolone) or CHOP‐like regimens (old regimen). The median number of chemotherapy cycles was three (range, 1‐9).

### 2.3 Statistical analysis

Overall survival was measured from the date of first treatment to the date of last follow‐up or death of any cause. Progression‐free survival (PFS) was measured from the first treatment to the date of relapse or progression, last follow‐up, or death of any cause. PFS at 12 or 24 months was defined as progression‐free disease at 12 or 24 months after treatment. Survival was estimated by the product‐limit Kaplan‐Meier method and compared using the log‐rank test. Propensity score analysis with 1:2 matching (PSM) was performed with the nearest neighbor matching method to pair each patient receiving chemotherapy alone and radiotherapy whose propensity score was within the designated caliper size.

To evaluate curability, we compared the mortality of elderly early‐stage NKTCL (observed) patients with the general Chinese population using age‐, sex‐, and calendar period‐generated (expected) mortality rates from the National Bureau of Statistics of the People's Republic of China.^30^ Observed vs expected survival was plotted using a conditional approach^31^ and expressed as standardized mortality ratios (SMR) of observed‐to‐expected deaths in R using the general Chinese population.^32^ All statistical data analyses were performed using IBM SPSS Statistics, version 24.0 and R v3.4.2. PSM was performed using the Matchit package in R.^33^

## 3 RESULTS

### 3.1 Patient characteristics

Patient characteristics and survival are listed in Table 1. Median patient age was 66 years old (range 61‐87). Most patients (n = 219, 68.2%) were <70 years old, 92 patients (28.7%) were 70‐79 years old, and 10 patients (3.1%) were >80 years old. Elderly patients were predominately male, with a male:female ratio of 3.5:1. Most patients exhibited good performance status and primary disease at the UADT site. Elevated lactate dehydrogenase (LDH) and B symptoms were observed in <30% of patients. Primary tumor invasion (PTI) was present in 52.0% of patients, and the majority of patients presented with stage I disease (71.0%).

**Table 1:** Univariate analysis of the association between clinical characteristics and survival outcomes in elderly patients with early‐stage NKTCL

Characteristic	No. (%)	5‐year OS		5‐year PFS	
		%	*P*	%	*P*
Sex					
Male	249 (77.6)	55.7	0.211	50.2	0.293
Female	72 (22.4)	67.2		58.1	
B symptoms					
No	229 (71.3)	60.6	0.069	54.4	0.100
Yes	72 (22.4)	51.7		45.5	
Elevated LDH					
No	232 (72.3)	61.3	0.069	54.9	0.124
Yes	89 (27.7)	50.1		45.0	
ECOG PS					
0‐1	292 (91.0)	60.8	0.002	54.0	0.001
≥2	29 (9.0)	34.7		32.9	
PTI					
Absence	154 (48.0)	72.7	<0.001	63.7	0.001
Presence	167 (52.0)	44.5		40.9	
Ann Arbor stage					
I	228 (71.0)	63.3	0.001	54.4	0.005
II	93 (29.0)	44.6		47.8	
Risk group					
0 (low risk)	99 (30.8)	80.0	<0.001	71.8	<0.001
≥1 (high risk)	222 (69.2)	47.9		43.1	

NKTCL, extranodal nasal‐type NK/T‐cell lymphoma; OS, overall survival; PFS, progression‐free survival; LDH, lactate dehydrogenase; ECOG, Eastern Cooperative Oncology Group; PS, performance status; PTI, primary tumor invasion.

### 3.2 Risk stratification and prognosis

**Figure 1:**
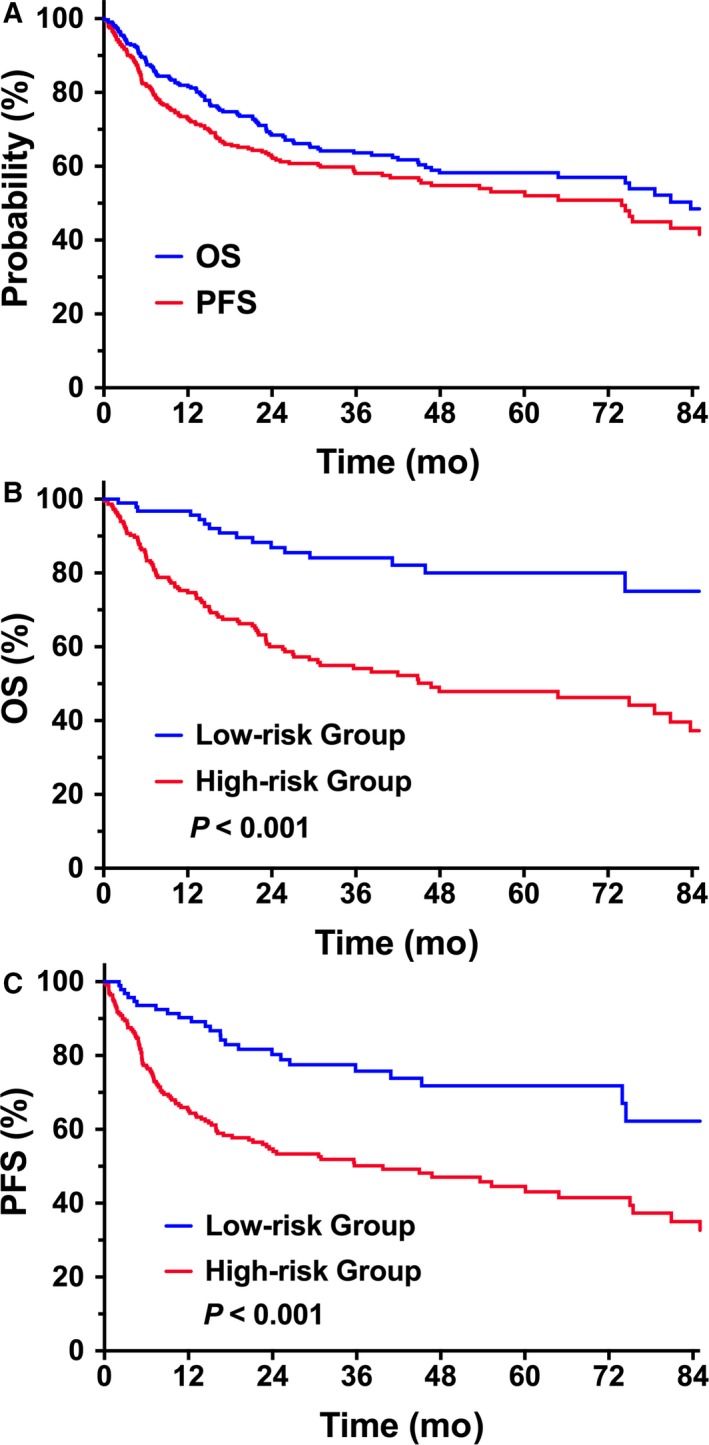
OS and PFS of elderly patients with early‐stage NKTCL. (A) OS and PFS in all patients. (B) OS and (C) PFS of elderly patients stratified into low‐ and high‐risk groups

The prognostic significance of clinical features for survival was evaluated for all patients (Table 1). In the univariate analysis, PS, PTI, and stage all significantly influenced OS and PFS. With a median follow‐up of 42 months for living patients, 5‐year OS and PFS for all patients were 58.2% and 53.1%, respectively (Figure 1A).

Modified from our previous nomogram model,^25^ elderly patients with early‐stage NKTCL were stratified into low‐ or high‐risk groups based on four prognostic factors [ECOG (Eastern Cooperative Oncology Group) score ≥2, stage II disease, elevated LDH, PTI], not including age >60 years. Patients without risk factors (0 risk factor) were defined as low risk (n = 99), and those with any risk factor (≥1 risk factor) were defined as high risk (n = 222). Low‐risk patients showed significantly greater OS and PFS than did high‐risk patients. Five‐year OS and PFS were 80.0% and 71.8%, respectively, in the low‐risk group compared with 47.9% (*P < *0.001, Figure 1B) and 43.1% in the high‐risk group (*P < *0.001, Figure 1C), respectively. Similarly, for the 262 patients treated with radiotherapy, we observed a significant difference in survival between the two risk groups, with a 5‐year OS and PFS of 82.2% and 74.4%, respectively, in the low‐risk group (n = 87), and 50.4% (*P < *0.001) and 47.5% (*P < *0.001) in the high‐risk group (n = 175), respectively.

### 3.3 Improved survival of radiotherapy vs chemotherapy alone

**Figure 2:**
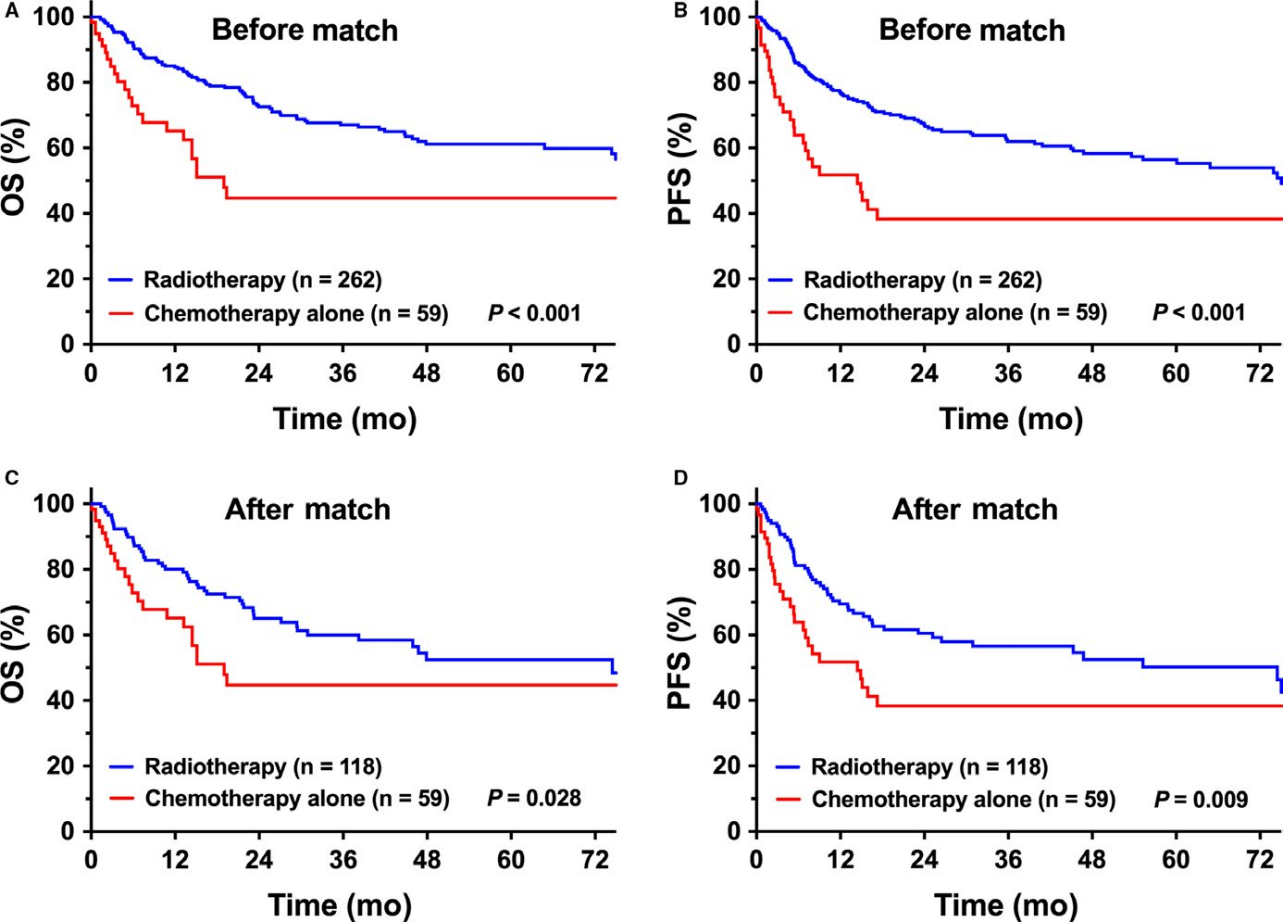
Comparison of OS and PFS between radiotherapy and chemotherapy treatments. (A) OS and (B) PFS of elderly patients with early‐stage NKTCL after single radiotherapy vs chemotherapy treatment before match stratification. (C) OS and (D) PFS of elderly patients with early‐stage NKTCL after single radiotherapy vs chemotherapy treatment after match stratification

We first evaluated the efficacy of individual radiotherapy vs chemotherapy treatment in elderly patients. Patients treated with chemotherapy alone tended to have more adverse factors (B symptoms, PTI, and stage II) than those treated with radiotherapy tended to have. The treatment outcome for radiotherapy in elderly patients was more favorable than that of chemotherapy alone (Table 2). The unadjusted 5‐year OS and PFS were 44.7% and 38.3%, respectively, for chemotherapy alone (n = 59) compared to 61.2% (*P < *0.001, Figure 2A) and 56.4% (*P < *0.001, Figure 2B), respectively, for radiotherapy (n = 262). There was no significant difference in survival between CMT and radiotherapy alone. The 5‐year OS and PFS were 58.1% and 53.0% for CMT, respectively, and 66.9% (*P* = 0.463) and 62.8% (*P* = 0.570), respectively, for radiotherapy alone.

**Table 2:** Clinical characteristics of elderly patients with early‐stage NKTCL before and after PSM stratification by treatment

Characteristic	Before PSM			After PSM		
	RT	CT alone	*P*	RT	CT alone	*P*
	No. (%)	No. (%)		No. (%)	No. (%)	
Total	262	59		118	59	
Sex						
Male	203 (77.5)	46 (78.0)	0.936	98 (83.1)	46 (78.0)	0.413
Female	59 (22.5)	13 (22.0)		20 (16.9)	13 (22.0)	
B symptoms						
No	198 (75.6)	31 (52.5)	<0.001	63 (53.4)	31 (52.5)	0.915
Yes	64 (24.4)	28 (47.5)		55 (46.6)	28 (47.5)	
Elevated LDH						
No	195 (74.4)	37 (62.7)	0.069	75 (63.6)	37 (62.7)	0.912
Yes	67 (25.6)	22 (37.3)		43 (36.4)	22 (37.3)	
ECOG PS						
0‐1	241 (92.0)	51 (86.4)	0.180	107 (90.7)	51 (86.4)	0.391
≥2	21 (8.0)	8 (13.6)		11 (9.3)	8 (13.6)	
PTI						
Absence	133 (50.8)	21 (35.6)	0.035	46 (39.0)	21 (35.6)	0.661
Presence	129 (49.2)	38 (64.4)		72 (61.0)	38 (64.4)	
Ann Arbor stage						
I	194 (74.0)	34 (57.6)	0.012	74 (62.7)	34 (57.6)	0.513
II	68 (26.0)	25 (42.4)		44 (37.3)	25 (42.4)	

NKTCL, extranodal nasal‐type NK/T‐cell lymphoma; PSM, propensity score‐matched; RT, radiotherapy; CT, chemotherapy; LDH, lactate dehydrogenase; ECOG, Eastern Cooperative Oncology Group; PS, performance status; PTI, primary tumor invasion.

After adjusting for confounding variables via PSM, all clinical features were balanced between radiotherapy and chemotherapy alone groups (Table 2). Radiotherapy resulted in better survival than chemotherapy alone. Adjusted 5‐year OS and PFS were 52.5% and 50.2%, respectively, for radiotherapy (n = 118) compared with 44.7% (*P = *0.028, Figure 2C) and 38.3% (*P = *0.009, Figure 2D), respectively, for chemotherapy alone (n = 59).

### 3.4 Survival benefit of new chemotherapy regimens

**Figure 3:**
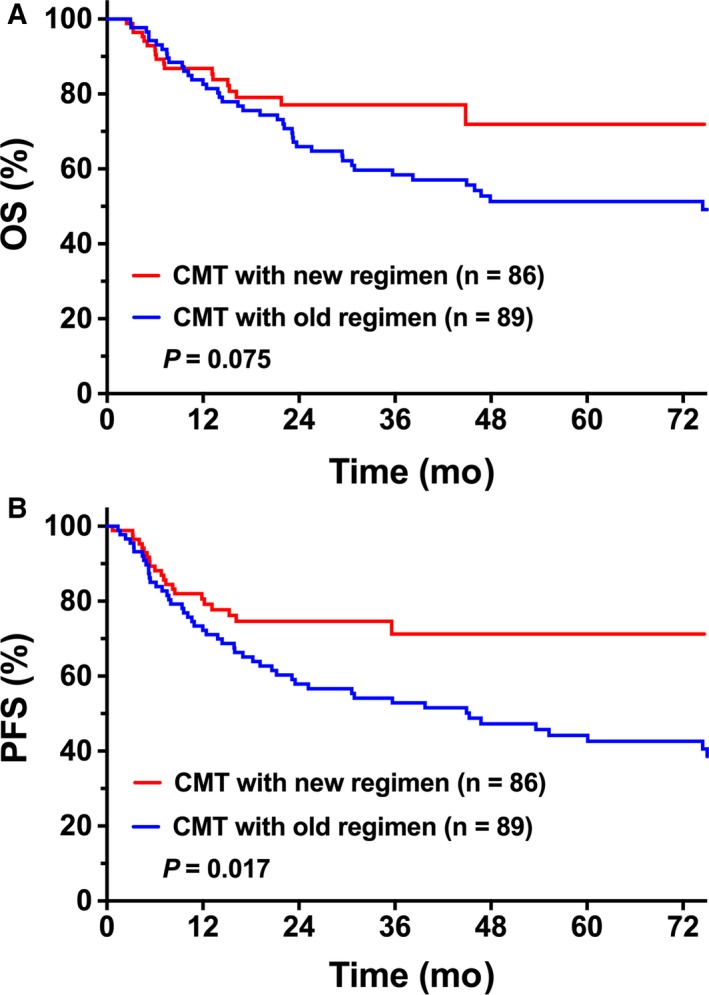
Comparison of OS and PFS between new and old chemotherapy regimens and radiotherapy. (A) OS and (B) PFS of elderly patients with early‐stage NKTCL who received the new chemotherapy regimen vs the old regimen and radiotherapy

We compared survival outcomes between new and old regimens of chemotherapy in the setting of CMT. Patients treated with the new CMT regimen showed significantly improved PFS and demonstrated a trend toward improved OS compared with the old regimen. Five‐year OS and PFS were 71.9% and 71.2%, respectively, for the new CMT regimen (n = 89) compared to 51.3% (*P* = 0.075, Figure 3A) and 44.2% (*P* = 0.017, Figure 3B), respectively, for the old CMT regimen (n = 86). However, there was no significant difference in OS (*P* = 0.574) or PFS (*P* = 0.363) between the new CMT regimen and radiotherapy alone.

### 3.5 Risk‐dependent curability in response to radiotherapy in comparison with the general population

**Figure 4:**
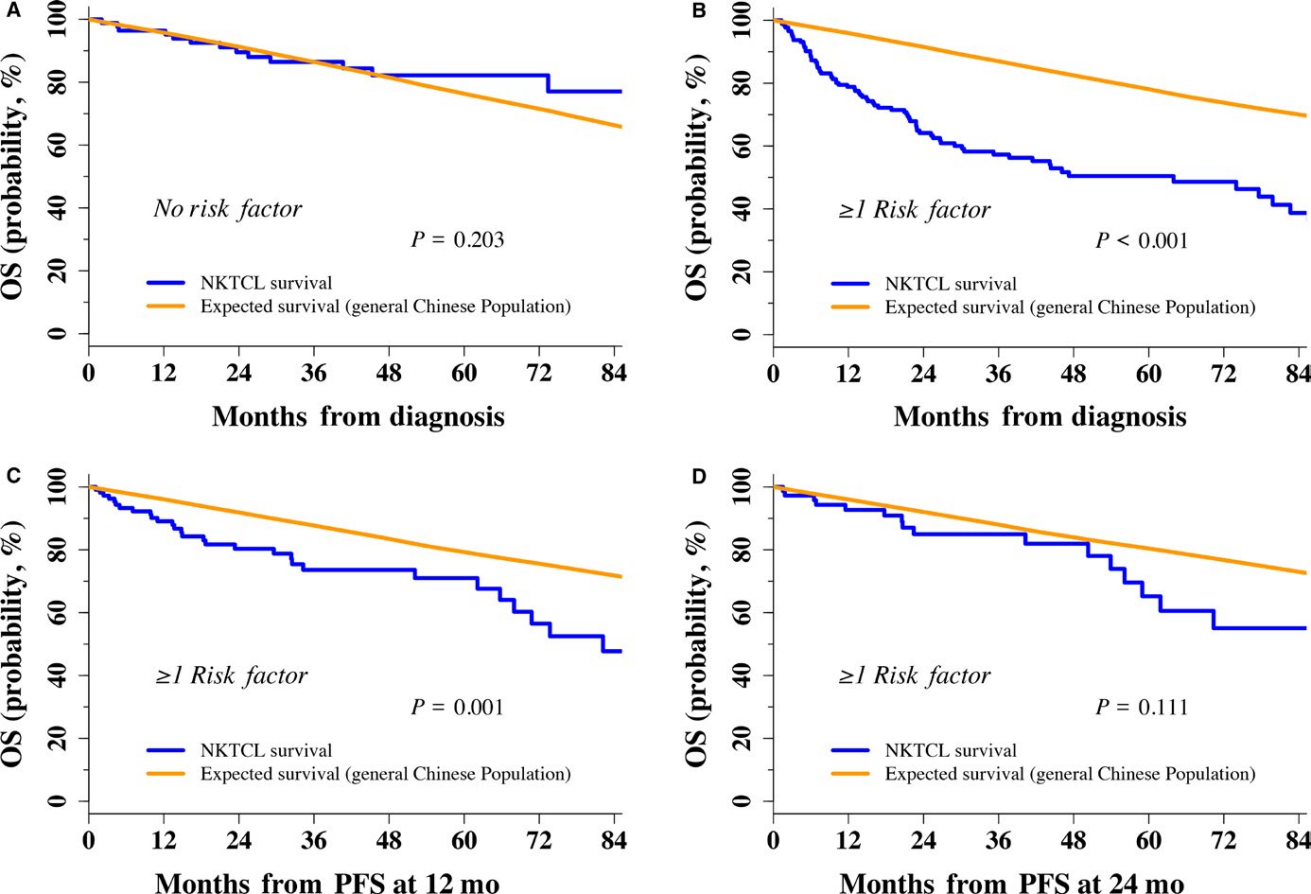
Relative OS of elderly early‐stage patients treated with radiotherapy compared with the general Chinese population. (A) Initial treatment of low‐risk patients (n = 87). (B) Initial treatment of high‐risk patients (n = 175). (C) PFS at 12 months in high‐risk patients (n = 111). (D) PFS at 24 months in high‐risk patients (n = 77)

To further determine the curability of early‐stage NKTCL in response to radiotherapy in elderly patients, SMR was used to quantify the decrease in mortality of the study cohort vs the general population. Observed OS in risk‐stratified elderly patients and expected OS in the matched general Chinese population are presented in Figure 4. Low‐risk patients treated with radiotherapy showed a very favorable survival equivalent to the general population at initial treatment, with an SMR of 0.703 (95% CI, 0.408‐1.210; *P = *0.203; Figure 4A). However, loss of life was pronounced in high‐risk patients treated with radiotherapy but was reduced for these patients achieving PFS at 12 or 24 months. Baseline SMR of high‐risk patients was 3.191 (95% CI, 2.556‐3.984; *P < *0.001, Figure 4B), indicating that high‐risk patients had, on average, triple the risk of death compared to the general population. High‐risk patients achieving PFS at 12 months exhibited significantly decreased survival compared to the general population, with an SMR of 1.867 (95% CI, 1.298‐2.687; *P = *0.001, Figure 4C). Eventually, high‐risk patients achieving PFS at 24 months had subsequent survival comparable to the general population, with an SMR of 1.490 (95% CI, 0.913‐2.431; *P = *0.111, Figure 4D). This finding indicates that radiotherapy cured a subset of elderly patients with early‐stage NKTCL and is risk‐dependent.

### 3.6 Death and safety

At last follow‐up, 114 patients (35.5%) had died, 93 (29.0%) due to lymphoma, 13 (4.0%) due to other diseases, and eight (2.5%) from treatment‐related complications. The eight latter patients died of chemotherapy‐related toxicities, including infection or failures of the liver, kidney, and/or heart. There was no significant difference in chemotherapy‐related deaths between non‐anthracycline‐based chemotherapy (5/122, 4.1%) and anthracycline‐based chemotherapy (3/112, 2.7%; *P* = 0.356). However, no patients died of radiotherapy‐related toxicity or developed secondary neoplasms.

## 4 DISCUSSION

The prognosis and role of radiotherapy or new chemotherapy regimens for elderly patients with early‐stage NKTCL have not been well defined. The present study represents the largest series of elderly early‐stage patients ever reported in a real‐world context and confirmed that radiotherapy achieves a favorable outcome and is significantly better than chemotherapy alone. New chemotherapy regimens combined with radiotherapy provide better survival than old regimens. Elderly early‐stage patients can be discriminated into low‐ and high‐risk groups with significantly different survival rates. When treated with radiotherapy, low‐risk patients at treatment and high‐risk patients achieving progression‐free survival at 24 months showed favorable outcomes, with survival equivalent to that of the general Chinese population. These findings highlight the risk‐dependent curability of early‐stage NKTCL in response to radiotherapy and provide a risk‐adapted follow‐up and counsel scheme for elderly patients.

Studies have demonstrated that older patients with lymphoma experience attenuated survival.^11^, ^12^, ^13^, ^15^ The prognosis of older patients with NKTCL is poorer than that of childhood patients.^18^, ^19^ In recently developed prognostic models for NKTCL, older age (>60 years old) has been identified as an adverse prognostic factor.^8^, ^25^ Our previous study developed and validated a nomogram model based on five independent prognostic factors for NKTCL.^5^ In the present study, using an age‐adjusted risk category system according to four risk factors (elevated LDH, ECOG score ≥2, stage II, and PTI) not including >60 years old, elderly patients with early‐stage NKTCL were successfully stratified into low‐risk (0 risk factor) or high‐risk (≥1 risk factor) groups, exhibiting a significant difference in 5‐year OS (80.0% vs 47.9%, respectively) and PFS (71.8% vs 43.1%, respectively). This risk stratification offers important indications for patient treatment, counseling, and prognostication.

The effect of older age on prognosis and treatment is not clearly defined for elderly patients with NKTCL.^8^, ^25^, ^34^ Due to the rarity and heterogeneity of clinical characteristics and management, elderly patients present with a variety of treatment outcomes.^19^, ^20^, ^21^ In this large multicenter cohort study of elderly early‐stage patients, we demonstrated that primary radiotherapy achieved a favorable outcome with a 5‐year OS of 61.2%; however, chemotherapy alone conferred an unfavorable prognosis (OS of 44.7%). These findings are consistent with previous studies comparing survival between individual radiotherapy and chemotherapy treatment in adult patients with early‐stage NKTCL.^10^, ^25^ Similarly, in another single institution study of 48 elderly early‐stage patients,^20^ radiotherapy conferred significantly better 5‐year OS than chemotherapy alone (55.3% vs 18.0%, *P* < 0.001). These findings demonstrate that radiotherapy plays a key role in the treatment of elderly patients with early‐stage NKTCL. In contrast, poor prognosis reported in other studies (5‐year OS <50%) may be attributed to selection bias with very small patient numbers,^19^ heterogeneity in clinical characteristics, or inadequate therapy with chemotherapy alone or low radiotherapy dose.^10^, ^21^

Several studies have shown that new chemotherapy regimens improve survival for NKTCL.^9^, ^20^, ^23^, ^24^ However, treatment outcomes vary widely, and most patients experienced severe toxicities. Prospective clinical trials evaluating the efficacy and toxicity of new chemotherapy regimens usually exclude the elderly. It is not clear whether intensified new chemotherapy regimens for young patients are appropriate for elderly patients when treated with combined radiotherapy and chemotherapy. In the current study examining elderly patients with early‐stage NKTCL, we demonstrated that non‐anthracycline‐based chemotherapy and radiotherapy conveyed significantly better PFS than anthracycline‐based chemotherapy and radiotherapy without significantly increasing the risk of death. A similar favorable outcome was observed in a small series of 14 elderly early‐stage patients treated with asparaginase‐based chemotherapy and radiotherapy.^20^ Further study is required to determine the efficacy and toxicity of incorporating more effective chemotherapy regimens and radiotherapy in risk‐stratified patients.

Normalization to the survival of a matched general population provides an important alternative to classic time‐to‐event analysis for curability of radiotherapy. Interestingly, elderly patients with low‐risk early‐stage NKTCL treated with radiotherapy in our study showed survival similar to the age‐ and sex‐matched general Chinese population. However, high‐risk early‐stage patients were at a 1.8‐ to 3.2‐fold higher risk of mortality within the first two years, but patients who were progression free at 24 months had subsequent survival comparable to that of the general population. Based on this pivotal study, this specific risk‐stratified benefit suggests that it is possible to obtain long‐term disease control with radiotherapy and have a real chance of curing a subset of elderly patients with early‐stage NKTCL. A similar phenomenon was observed in patients with DLBCL after immunochemotherapy.^35^, ^36^ Given a normal life expectancy in low‐risk patients with treatment and high‐risk patients who are progression free at 24 months, utilization of a routine intensified surveillance strategy may not be necessary for elderly early‐stage patients in remission beyond these time points.

Considering the frequency of comorbidities and diminished organ functions,^37^, ^38^ whether elderly patients with early‐stage NKTCL can tolerate intensified new chemotherapy regimens and high‐dose and large‐field radiotherapy is still an important topic of concern.^39^ Previous studies have reported that the use of anthracycline‐based regimens was associated with a high risk of severe hematologic toxicities in old cancer patients.^38^, ^40^ Furthermore, severe acute toxicities and treatment‐related death during non‐anthracycline‐based chemotherapy were common in patients with NKTCL.^23^, ^24^, ^41^ In contrast to DLBCL patients treated with involved‐site or involved‐node radiotherapy at moderate doses of 30‐40 Gy, NKTCL patients received extended involved‐site radiotherapy with a radical dose of at least 50 Gy.^26^, ^29^ In the present study, death due to chemotherapy occurred in 3.4% of elderly patients with early‐stage NKTCL, whereas no patients died of radiation‐related complications or secondary cancers. Best supportive care potentially plays an important role in the initial treatment of elderly patients with NKTCL. However, we did not observe a significant difference in chemotherapy‐related deaths between non‐anthracycline‐based and anthracycline‐based regimens. These findings highlight that radiotherapy is well tolerated in the elderly population and underscores the need to administer curative therapy, while minimizing treatment‐related toxicity. Physicians should balance the benefit and toxicity as well as consider risk‐stratified patients when choosing a treatment strategy.

There are several limitations because of the nature of this retrospective study. First, although the data confirm better survival after radiotherapy compared with chemotherapy alone or non‐anthracycline‐based regimens compared with anthracycline‐based regimens, the treatments were not randomly assigned. Therefore, the results may be affected by selection biases. We attempted to circumvent this limitation using PSM to account for prognostic factors. However, the survival benefit in elderly patients with early‐stage NKTCL needs to be confirmed in large, prospective studies. Secondly, because positron emission tomography (PET) was not routinely performed in this study, the staging bias could not be avoided. Further well‐designed study is required to draw firm conclusions regarding the optimal staging and treatment strategy for elderly patients.

In conclusion, radiotherapy achieves a favorable outcome for elderly patients with early‐stage NKTCL. Low‐risk patients in treatment and high‐risk patients who achieve PFS at 24 months show a subsequent OS equivalent to that of the general Chinese population. We demonstrate the efficacy and feasibility of radiotherapy and a new chemotherapy regimen in elderly patients with early‐stage NKTCL.

## CONFLICT OF INTEREST

The authors have declared no conflict of interests.
